# Direct Cortical Stimulation to Probe the Ictogenicity of the Epileptogenic Nodes in Temporal Lobe Epilepsy

**DOI:** 10.3389/fneur.2021.761412

**Published:** 2022-01-13

**Authors:** Auriana Irannejad, Ganne Chaitanya, Emilia Toth, Diana Pizarro, Sandipan Pati

**Affiliations:** ^1^Department of Neurology, University of Alabama at Birmingham, Birmingham, AL, United States; ^2^Epilepsy and Cognitive Neurophysiology Laboratory, University of Alabama at Birmingham, Birmingham, AL, United States

**Keywords:** direct cortical stimulation, seizure onset zone, ictogenicity, temporal lobe epilepsy, epileptogenicity

## Abstract

Accurate mapping of the seizure onset zone (SOZ) is critical to the success of epilepsy surgery outcomes. Epileptogenicity index (EI) is a statistical method that delineates hyperexcitable brain regions involved in the generation and early propagation of seizures. However, EI can overestimate the SOZ for particular electrographic seizure onset patterns. Therefore, using direct cortical stimulation (DCS) as a probing tool to identify seizure generators, we systematically evaluated the causality of the high EI nodes (>0.3) in replicating the patient's habitual seizures. Specifically, we assessed the diagnostic yield of high EI nodes, i.e., the proportion of high EI nodes that evoked habitual seizures. A retrospective single-center study that included post-stereo encephalography (SEEG) confirmed TLE patients (*n* = 37) that had all high EI nodes stimulated, intending to induce a seizure. We evaluated the nodal responses (true and false responder rate) to stimulation and correlated with electrographic seizure onset patterns (hypersynchronous-HYP and low amplitude fast activity patterns-LAFA) and clinically defined SOZ. The ictogenicity (i.e., the propensity to induce the patient's habitual seizure) of a high EI node was only 44.5%. The LAFA onset pattern had a significantly higher response rate to DCS (i.e., higher evoked seizures). The concordance of an evoked habitual seizure with a clinically defined SOZ with good outcomes was over 50% (*p* = 0.0025). These results support targeted mapping of SOZ in LAFA onset patterns by performing DCS in high EI nodes to distinguish seizure generators (true responders) from hyperexcitable nodes that may be involved in early propagation.

## Introduction

Intracranial EEG investigation aims to localize seizure generators and, in resection cases, to define the anatomical extent of surgical resection that will maximize the chance of seizure freedom. The epileptogenic zone (EZ) is conceptualized as the area of the cortex that is indispensable for the generation of epileptic seizures, the removal of which would contribute to seizure freedom (1) Increasingly, the EZ is considered a network of functionally interconnected structures that can involve anatomically non-contiguous regions (2, 3). In temporal lobe epilepsy (TLE), the epileptogenic network (EN) can extend beyond the mesial temporal structures to include nearby extra-temporal regions such as the orbitofrontal cortex. Failure to identify and resect these extra-temporal structures (known as TLE-plus) is associated with seizure recurrence following anterior temporal lobectomy (4–6). Thus, there is a clinical need to develop imaging or electrophysiological parameters (or biomarkers) to delineate the full extent of the EN preoperatively to optimize the surgical outcome.

Stereoelectroencephalography (SEEG) allows high-resolution mapping of candidate biomarkers of epileptogenicity and offers insights into pathophysiological processes within the EN (5). Both lower and higher-frequency neural activities (infra slow- and high-frequency oscillations) and the epileptogenicity index (EI) are some of the parameters used to map the EN (7–11). Specifically, the EI statistically summarizes the spectro-temporal parameters of SEEG signals at seizure genesis and is related to the propensity of a brain area to generate low voltage fast discharges (12). Thus, the EI can be used to quantify the epileptogenicity of brain structures in the early organization of seizure genesis with an index ranging from 0 (no epileptogenicity) to 1 (maximal epileptogenicity).

However, there are a few challenges in the clinical interpretation of the EI. First, the EI does not distinguish nodes involved in the initiation vs. early propagation of seizures (5). Second, the estimation of EI is susceptible to the imperfect spatial sampling that is inherent to any invasive EEG, including SEEG. For example, the low voltage fast discharges at seizure onset can present quasi-simultaneously over a vast territory that may overestimate the EN, or the seemingly first electrographic changes may represent propagated ictal activity, thereby false localizing the EN. Direct cortical stimulation (DCS) to evoke and replicate a patient's habitual aura offers an alternative strategy to probe the putative epileptogenic nodes and delineate the EN that is indispensable for seizure generation (13, 14).

Prior studies have validated the EI with clinically identified seizure onset zone (3), interictal high-frequency oscillation maps (15), and post-resection seizure outcome (5), but none to date have correlated the EI with DCS. In the present study, using DCS as a probing tool to identify seizure generators, we systematically evaluated the causality of the high epileptogenicity index nodes (>0.3) in replicating the patient's habitual seizures. Specifically, using DCS in a cohort of highly selected patients, we evaluated the diagnostic yield of high EI nodes, i.e., the proportion of high EI nodes that evoked habitual seizures. We hypothesized that high EI nodes overlapped with the clinically defined seizure onset zone would yield the highest in evoking habitual seizures.

## Methods

### Patient Selection and Study Design

**Figure 1:**
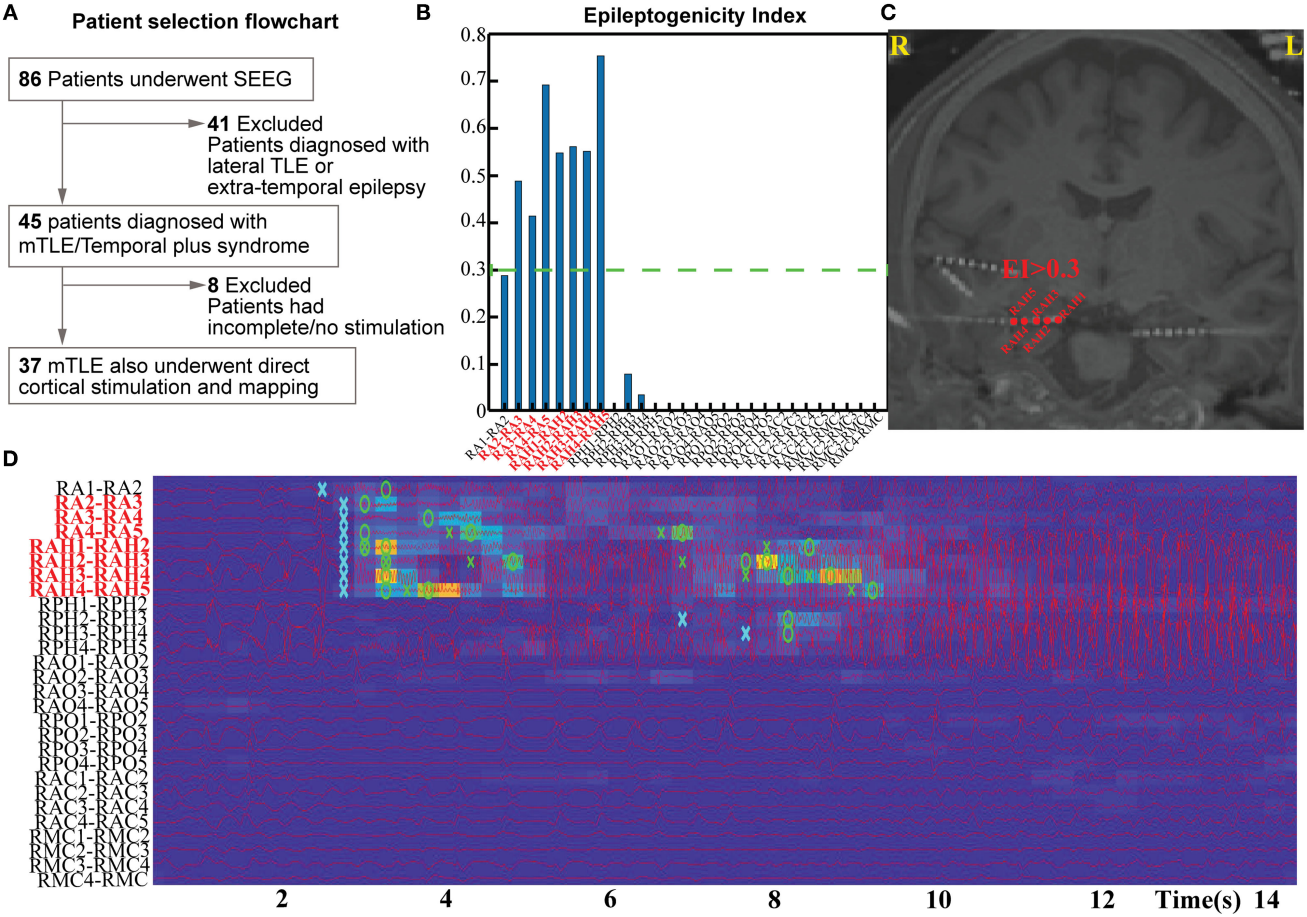
Patient selection flow chart **(A)** and estimation pipeline for Epileptogenicity Index (EI) for their seizures **(B–D)**.

We performed a single-center, retrospective study at a level-IV epilepsy center using protocols approved by the University of Alabama Birmingham Institutional Review Board. Since the inception of SEEG investigation at our center, eighty-six adults (>19-years old) with drug-resistant focal epilepsy have undergone the procedure successfully between January 2014 and January 2020. Forty-five of them had confirmed mesial TLE or TLE-plus epilepsies (the “plus” indicates additional seizure foci in neighboring regions, such as the insula, the suprasylvian operculum, the orbito-frontal cortex, and the temporo-parietooccipital junction) were included in this study (4, 16) (Figure 1A). The localization of the seizure onset zone (SOZ) was confirmed based on consensus among the multidisciplinary epilepsy group after reviewing the anatomical and electroclinical findings. DCS was performed as a part of the clinical protocol by the epileptologist investigating the patient. The inclusion criteria for the retrospective study were: (a) confirmed TLE or TLE plus epilepsies, (b) DCS performed on a significant proportion of sampled electrodes in clinically identified onset zones and propagated regions, including all of the high EI nodes. Patients were excluded if DCS was not performed on all of the high EI nodes.

### Stereoencephalography Surgery and Data Acquisition

SEEG electrodes were implanted into predetermined regions of interest for seizure localization using a robotic platform (ROSA® robot, Medtech, Montpelier). SEEG was recorded with cylindrical intracranial electrodes (0.8 mm outer diameter) with 5–20 contacts per electrode. Each contact was 2 mm in length with 1.5 mm intercontact distance (PMT® Corporation, Chanhassen, MN). The localization of the electrodes was confirmed using AAL2 atlas and iElectrodes toolbox (17, 18). Clinically defined seizure onset channels, along with contacts localized to gray matter, were parsed to reconstruct bipolar derivatives for subsequent estimation of EI. Contacts in white matter were not used for the analysis of EI. Intracranial video-EEG was sampled at 2048 Hz (Natus Medical Incorporated, Pleasanton, CA). An extracranial electrode common to all was placed posteriorly in the occiput near the hairline as the reference signal.

### Estimation of Epileptogenicity Index

The EI was used to quantify the epileptogenicity of brain structures. The EI delineates regions (or nodes) of the recorded brain activity involved in the generation of a rapid discharge (12) (Figures 1B–D). The energy spectral density ratio (ER) was estimated as a measure of an abrupt increase in fast oscillations in the SEEG signal [formula: ER = (*E*_12−127*Hz*_)/(*E*_4−12*Hz*_)]. The cumulative sum algorithm by Page and Hinkley helped improve the time point of detection of fast oscillations. EI was therefore calculated as the averaged ER overtime immediately following detection of a rapid discharge in the first channel divided by the delay of involvement across other channels. The EI values were computed for all the channels identified in the gray matter. The first 20 s of the seizure were analyzed with −10 s to +10 s segment selected around the seizure onset as determined by epileptologists. For a channel to be considered within the epileptogenic network—and subsequently involved seizure onset channels—they had to demonstrate an average EI value above 0.3 (calculated from all available seizures per patient). Multiple seizures (2–8) were analyzed per subject to estimate the EI.

### Electrographic Patterns at Seizure Onset

Electrographic seizure onset patterns have been associated with various epileptogenic lesions, distribution of high-frequency oscillations, and surgical outcomes (19–21). Although a repertoire of electrographic onset patterns has been reported, we restricted the seizure onset patterns to three predominant types due to the limited sample size. These patterns were: hypersynchronous patterns (HYP), low amplitude fast activity (LAFA), or mixed if they had both LAFA and HYP features intermixed at the seizure onset.

### Direct Cortical Stimulation

DCS was performed (Nicolet® stimulator) by the epileptologist responsible for the surgical evaluation of the patient while admitted to the epilepsy monitoring unit (EMU). At the time of DCS, the clinician was unaware of the EI-identified nodes. The stimulation was performed toward the end of the EMU admission after spontaneous seizures were recorded and anti-seizure drugs were resumed. At our institute, stimulation is performed between bipolar channels at stimulation frequencies of either 50 or 1 Hz. Pulses were biphasic with pulse-widths ranging between 200 and −400 μ s. The stimulation trial begins with a brief survey of increasing current amplitudes (range 1–8 mA) tested in one or two brain regions (always remote to the seizure onset sites) to evaluate the threshold for after-discharges. Once the threshold is determined, the current strength for the subsequent trials is kept relatively unchanged. The most common current strength ranged between 3 and 5 mA (median 4 mA) that was delivered for 3–4 s for 50 Hz trials and 10 s for 1 Hz trials. Each stimulation session lasted between 45 min and 2 h, and in some patients, multiple sessions were performed over several days. The patient was awake during the stimulation, and the family was allowed to stay at the bedside. Parenteral lorazepam was available at the bedside to treat evoked seizures that were secondarily generalized. Video EEG of the stimulation sessions was archived for future reporting.

### Definition and Interpretation of Electroclinical Responses to DCS

**Figure 2:**
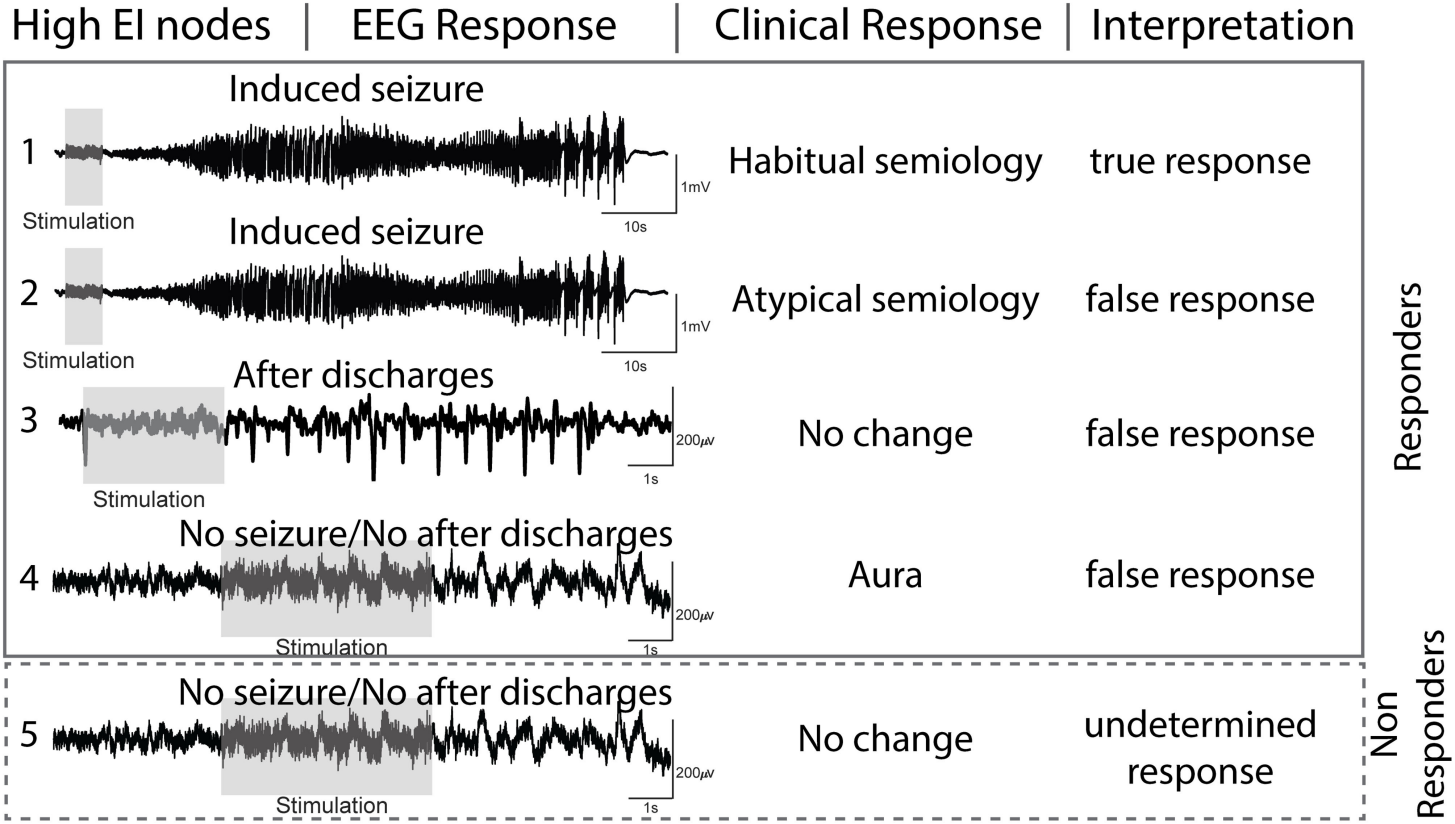
Interpretation of electrographic and clinical responses to direct cortical stimulation (DCS) of nodes with high epileptogenicity index (EI > 0.3).

The nodal responses to DCS can be summed by characterizing electrographic and clinical changes (Figure 2). The following are the definitions used in this study:

(a) After-discharges (AD)- After discharges were defined as rhythmic discharges (spikes, poly-spikes, sharp waves, or spike-wave complexes), which were clearly distinct from the pre-stimulation electrographic activity and occurred immediately following DCS (22). Any clinical symptoms did not accompany the discharges.
(b) Seizures (typical and atypical)- DCS-induced seizures were defined as trains of AD's that evolved morphologically, spatially, and/or in frequency and were accompanied by clinical manifestations. If the patient or family members recognized the behavioral changes as similar to spontaneous seizures, then we defined them as a habitual seizure. All other evoked seizures, including electrographic seizures, were considered atypical. Semiology was classified as: focal aware seizures (FAS), focal impaired awareness seizures (FIAS), and focal to bilateral tonic-clonic seizures (FBTCS) (23).
(c) Clinical response only- The term was reserved for patient-reported symptoms (e.g., motor activity or unusual feeling) evoked with DCS that lacked any electrographic changes (AD or seizure).
(d) No response (NR)- With DCS, there was no AD or seizure, and the patient did not report any clinical symptoms.

Based on the above-mentioned definitions, we interpreted the evoked electroclinical responses of the high EI nodes to DCS as-

(a) true response (TR) when the high EI nodes evoked a habitual seizure.
(b) false-response (FR) when high EI nodes failed to evoke a habitual seizure but either (1) evoked AD's, (2) evoked an atypical seizure, or (3) evoked a clinical response only. Since seizures are an all-or-none phenomenon, the presence of AD'sa confirmed that the node was stimulated adequately but failed to evoke a seizure. The presence of a clinical response without electrographic changes also ruled out a seizure and was interpreted as a false response.
(c) undetermined response (UR): high EI nodes that failed to evoke any response (electrographic or clinical) to DCS were considered undetermined as one cannot confirm with certainty if the nodes were stimulated adequately. Lack of response to DCS can be due to suboptimal stimulation.

### Seizure Outcome

We used the Engel scale to classify the outcome of interventional therapy (resection, ablation, or neuromodulation) at the last clinic visit. The median range in clinical follow-up post-intervention was eleven months, and the range was between 5 months and 4.2 years. Engel class I indicated free of disabling seizures; Engel class II, rare disabling seizures; Engel class III, worthwhile improvement; and Engel class IV, no worthwhile improvement.

### Statistical Measures

Based on the nodal responses to DCS, the diagnostic yield of high EI nodes were estimated as

(a) Responder rate = TR + FR/ TR + FR + UR
(b) Non-responder rate = UR/TR + FR + UR
(c) True responder rate: TR/TR + FR
(d) False responder rate: FR/FR + TR

Chi-square statistical analysis with a significance set at *p* < 0.05 was performed to evaluate if a nodal response (vs. no response) was different for the two EEG onset patterns (LAFA vs. HYP). Fisher exact test was performed to assess the responses (true or false response) of the nodes localized within vs. outside the clinically defined seizure onset zone.

## Results

### Cohort Demographics

Thirty-seven patients (female = 23) with a median age of 37 years (range 19–63 y) met the inclusion criteria (Table 1). The total number of depth electrodes implanted was 460 (median 12, range 7–20). Eighteen subjects had a bilateral implant. The seizure onset regions were mesial TLE (amygdala, hippocampus) and TLE-plus (*N* = 13), where the seizure foci extended beyond the amygdala-hippocampus to the insula, superior temporal gyrus, or orbitofrontal regions. Eleven patients (30%) had an epileptogenic lesion (e.g., hippocampal sclerosis, focal cortical dysplasia) identifiable in the preoperative brain MRI. One-hundred and sixty seizures (median 5 per subject) were analyzed to identify the high EI nodes. Twelve patients underwent subsequent anterior temporal lobectomy, five had an extended temporal lobectomy, and seven had Responsive Neurostimulation (RNS) Therapy.

**Table 1:** Clinico-demographic characteristics of the patients.

**Patients**	***N*** **= 37**
Age (years)	38.13 ± 11.44
Gender (male: female)	14:23
Duration of epilepsy (years)	14.69 ± 11.68
**MRI pathology laterality**	
Normal	13
Bilateral	10
Right	10
Left	4
MRI pathology type (epileptogenic lesion)	11 (30%)
**Electrode implant laterality**	
Left	11
Right	8
Bilateral	18
Number of electrodes per patient [median (range)]	12 (7–20)
**SEEG seizure onset pattern**	
Hypersynchronous (HYP)	12
Low amplitude fast activity (LAFA)	12
Mixed	13
Number of seizures analyzed to measure EI [total (median, range)]	160 (5, 2–8)
**Stimulation protocol types (number of trials across all patients)**	
50Hz,5–6mA	8
50Hz,4–8mA	1
50Hz,4–6mA	12
50Hz,4–5mA	3
1Hz,5—mA	7
50Hz,4–7mA	2
50Hz,5–7mA	7
50Hz,6–7mA	2
50Hz,3–5mA	1
**SEEG seizure onset zone localization**	
TLE (hippocampus amygdala complex)	24
TLE+ (ictal changes beyond mesial temporal structures)	13
**Post SEEG therapy**	
Anterior temporal lobectomy	12
Extended anterior temporal lobectomy	3
Other resections (temporal pole resection, cingulate, OF)	3
RNS	10
Awaiting treatment or patient declined intervention Rx	7
Stimulated high EI nodes across all patients (total, range/patient)	112, 1–5 contacts/patient
Responsive contacts	92 (82%)
Non responsive contacts	20 (18%)
**Engel outcome for patients with resection (*****N*** **of patients)**	
Engel I	9
Engel II	8
Engel III	1
**Engel outcome for patients with RNS (*****N*** **of patients)**	
Engel I	0
Engel II	3
Engel III	7

*TLE, temporal lobe epilepsy; EI, epileptogenicity index; RNS, responsive neurostimulation; OF, orbitofrontal*.

### High EI Nodes That Responded to DCS

**Figure 3:**
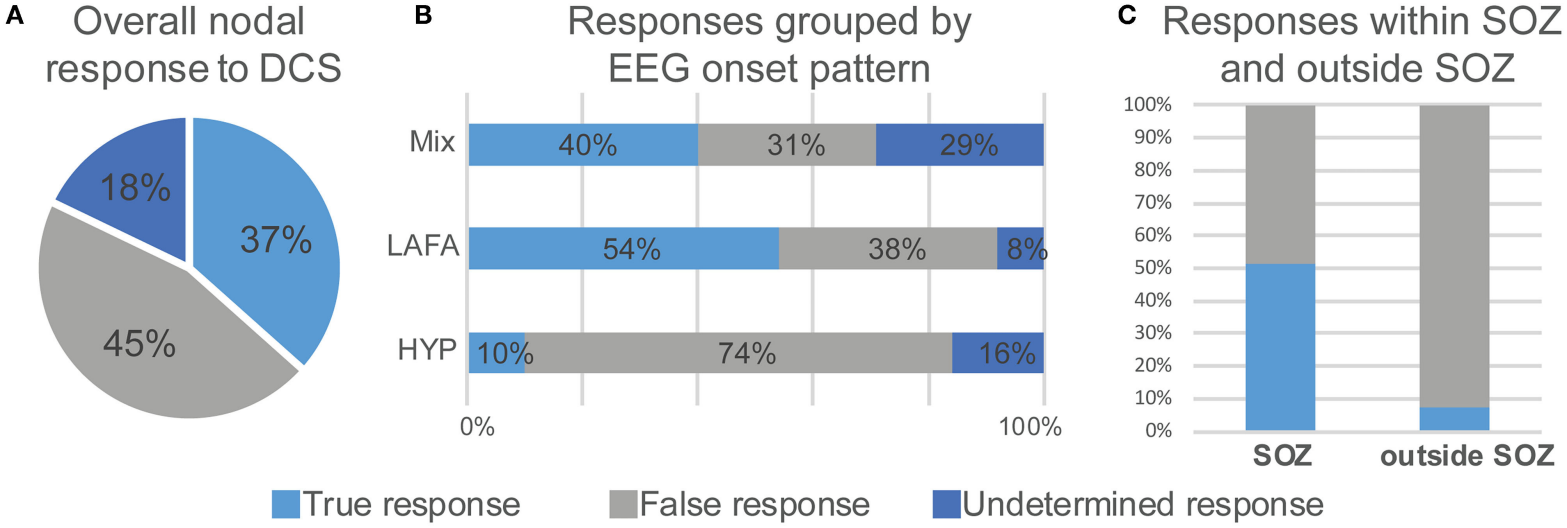
Electroclinical responses to direct cortical stimulation (DCS) of nodes with high epileptogenicity index (EI > 0.3). Overall nodal responses **(A)** and distribution of responses as a function of seizure onset pattern **(B)** and colocalization with seizure onset zone **(C)**. LAFA = low amplitude fast activity. HYP, hypersynchronous onset; SOZ, seizure onset zone.

Overall, in 37 patients, there were 112 high EI nodes (range 1–5 nodes per subject). Of the 112 stimulated nodes, 92 (82%) responded to DCS (Figure 3A). The remaining 20 (18%) were non-responders, i.e., they did not result in electrographic or clinical response to DCS. The LAFA pattern had a significantly higher responder rate than the HYP (*p* < 0.00001). Among the non-responders, the predominant electrographic onset patterns were mixed (*n* = 12, 60%) followed by HYP (*n* = 5) patterns (Figure 3B).

### High EI Nodes With a True Response to DCS

Forty-one nodes (37%) responded positively to DCS, i.e., evoked an electroclinical habitual seizure. The *true responder rate* of a high EI node was 44.5%. Among the true responders, 52% had LAFA, 41% had mixed, while 7% had a hypersynchronous pattern of seizure onset. Overall, there were 30 evoked FSA and 13 FIAS seizures. The concordance of an evoked habitual seizure with a clinically identified SOZ that had Engel I or II outcome was over 50% (*p* = 0.0025) (Figure 3C), and the regions were mostly hippocampus and amygdala (*N* = 12), although there were few insular and anterior cingulate regions (Table 1). These patients had a follow-up over 2–4 years after resection.

### High EI Nodes With a False Response to DCS

**Figure 4:**
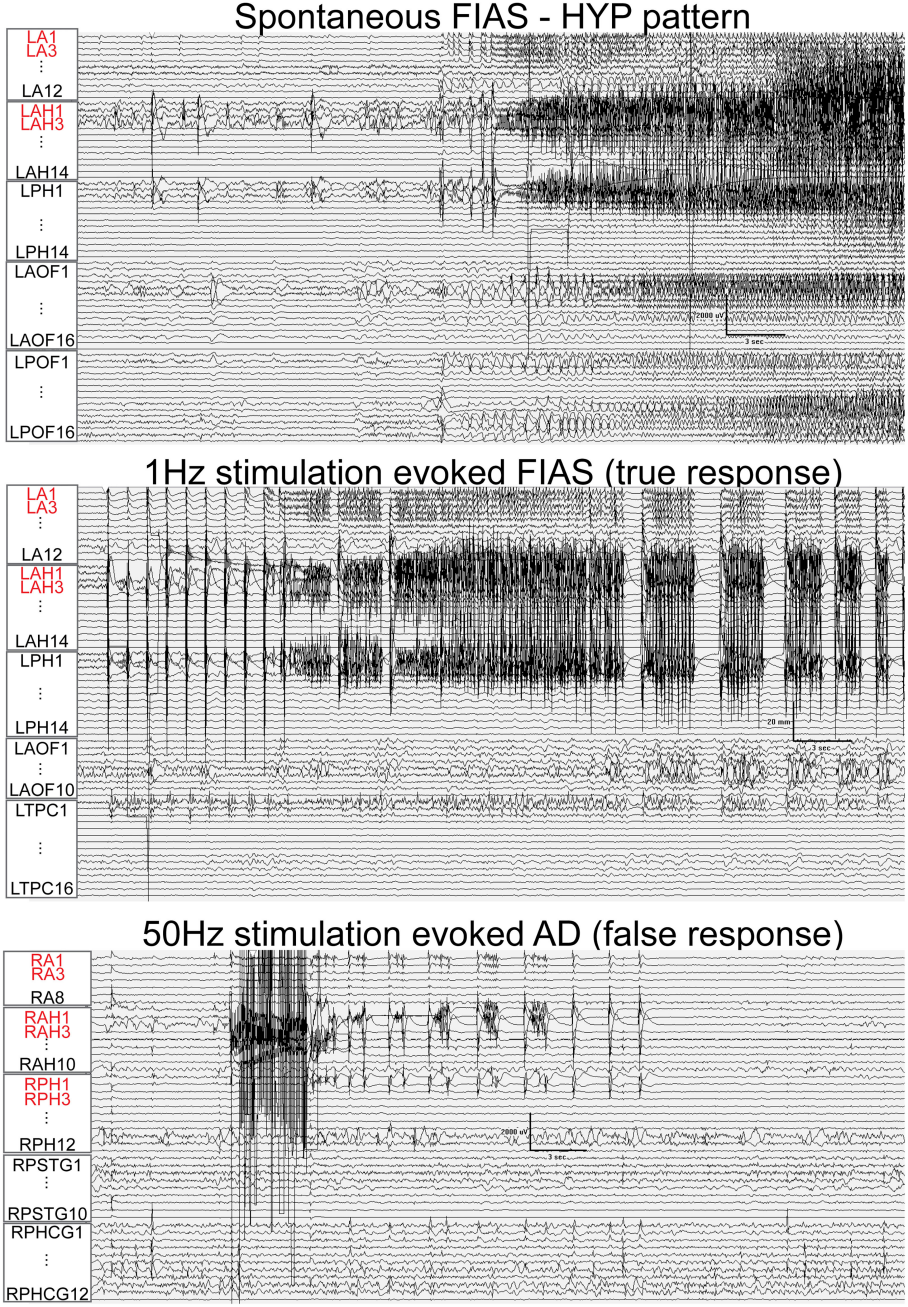
Train of 1 Hz and 50 Hz stimulation of hippocampus evoked seizure (true response) and after-discharges (AD, false responses) in different patients that had hypersynchronous (HYP) electrographic onset pattern of spontaneous seizure. Nodes with a high epileptogenicity index (>0.3) are highlighted in red. HYP, hypersynchronous pattern; FIAS, focal impaired awareness seizure.

**Figure 5:**
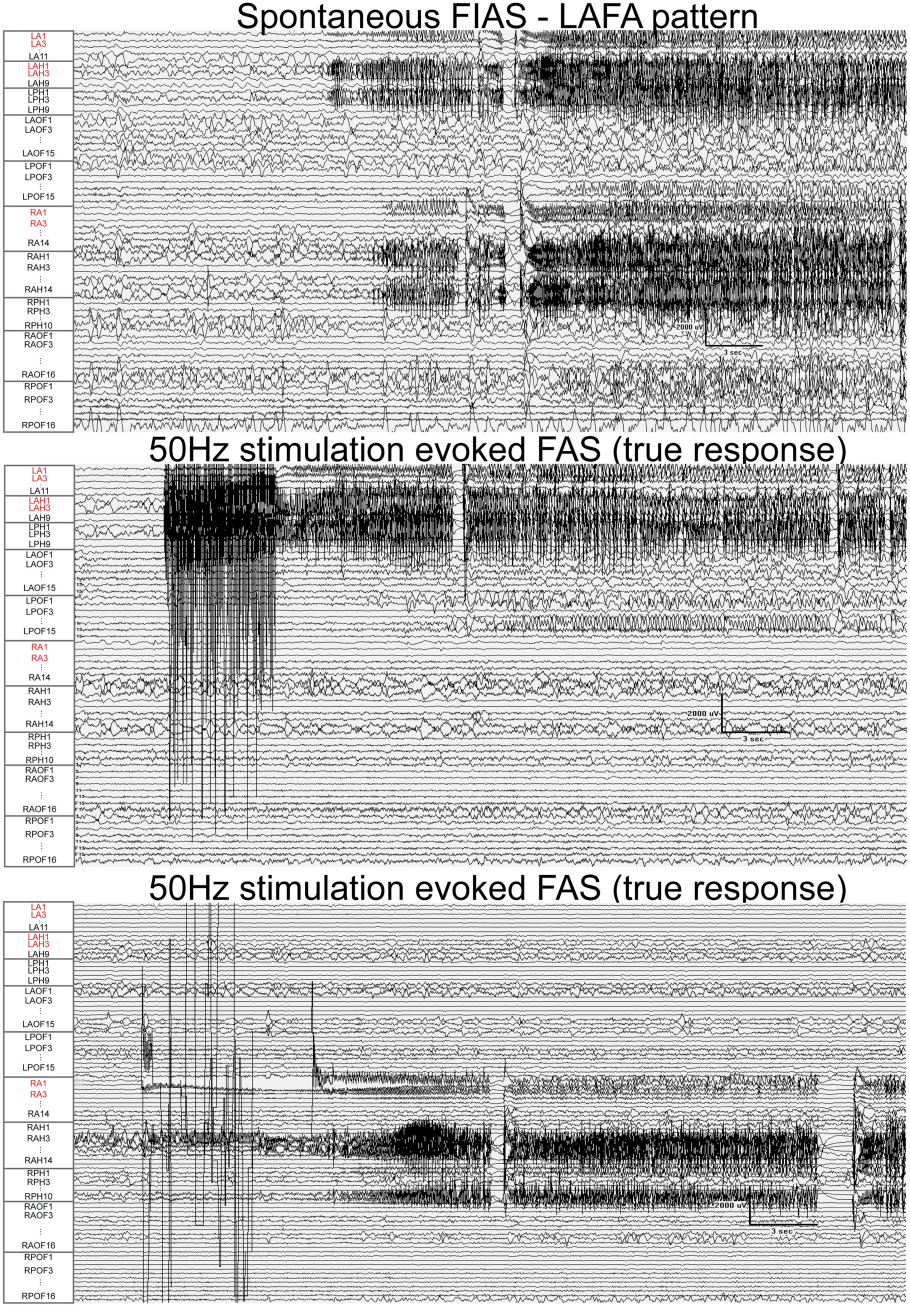
Fifty Hertz stimulation of left hippocampus and right amygdala evoked seizures (true responses) in a patient with bi-temporal epilepsy. Nodes with a high epileptogenicity index (>0.3) are highlighted in red. The spontaneous seizure had LAFA (low amplitude fast activity) pattern that emanated from the left hippocampus with a rapid propagation to the left amygdala, right amygdala, and hippocampus. FAS, focal seizure with retained awareness. FIAS, focal impaired awareness seizure.

Fifty-one nodes (45%) had a false response, with the presence of either AD's (*n* = 49), an atypical seizure (*n* = 1), or clinical symptoms without electrographic changes (*n* = 1). The *false responder rate* of a high EI node was 55.4. Hypersynchronous patterns (45%) yielded the maximum number of false responses, followed by LAFA (29.4%). After-discharges were the most common false responses (Figures 4, 5). The HYP pattern had ADs predominantly in the amygdala and hippocampus contralateral to clinical SOZ. For LAFA, the nodes were localized to the insula, posterior temporal, basal temporal, and lateral prefrontal regions.

## Discussion

DCS is a valuable tool in assessing the epileptogenic cortex and is essential in planning epilepsy surgery (24). DCS is used for functional mapping of the eloquent cortex and to delineate surgical resection margins by identifying hyperexcitable structures within the seizure generating network (25). Stimulation-induced seizures have been co-localized with spontaneous seizures, interictal pathological high-frequency oscillations and positively correlated with post-resection seizure-free outcomes (25–28). In the present study, we used DCS to investigate the ictogenicity of the putative epileptogenic nodes that had high EI values (>0.3). We demonstrated that the ictogenicity (i.e., inducing the patient's habitual seizure) of a high EI node is only 44.5%, while 55.4% failed to induce a seizure but had runs of after-discharges.

### Electrographic Onset Pattern Influenced Response to DCS

The LAFA onset pattern had a significantly higher responder rate to DCS (i.e., had a higher propensity to induce a seizure), while the HYP pattern yielded maximum false responses (i.e., had runs of AD's instead of seizures). The results are in agreement with a previous modeling study and underscore the differences in the mechanism of seizure genesis between the two patterns (29–31). The LAFA onset is initiated by the coalescence of multiple scattered regions of localized high-frequency activity over time (32). The EI overestimated the number of nodes for the LAFA pattern, and these nodes were localized outside the clinically identified SOZ, often in the temporal or frontal neocortex. Importantly, these nodes failed to evoke a seizure but had after-discharges. The HYP onset is characterized by an increase in the excitability of the surrounding tissue, which by itself does not generate seizures, but can support seizure activity. The lower ictogenicity and the higher false response rate of HYP in our study concur with the hypothesized mechanism.

### Probing High EI Nodes With DCS: A Translational Approach to Map Seizure Onset Network

The goal of intracranial EEG investigation is to delineate the brain regions that are involved in seizure generation, and this can be more challenging in MRI-normal non-lesional cases, typically necessitating a greater number of depth electrodes (median 12 in our cohort). DCS mapping of evoked seizures is an accepted approach to confirm the SOZ and co-localization have been positively correlated with good surgical outcomes. However, stimulating over 100–150 contacts is not feasible in routine clinical practice and is likely to be unpleasant for the patient. Rather, a targeted approach using DCS to probe high EI nodes can be more time-efficient and can distinguish hyperexcitable nodes involved in seizure generation (true responder) from nodes supporting early propagation (false responder). Such an approach in the future may also guide therapeutic decision-making by localizing more precise targets for laser therapy for a focal onset or facilitating placement of responsive neuromodulation stimulation electrodes.

### Study Limitations

The study had three major limitations. 1) The responses to DCS were only restricted to high EI nodes. Although DCS was performed in nodes with lower EI (<0.3), due to the heterogeneity in the sparse data, we could not perform meaningful statistics. An ideal study to assess EI's diagnostic yield (sensitivity, specificity) should include stimulation of a significant proportion of nodes (both low and high EI) in a large cohort prospectively. Planning such a study should also involve ethical approval as a patient-level of tolerance and safety should be considered. The second limitation is the inability to correlate true responsive nodes with the surgical outcome, as anterior temporal lobectomy (performed in 38% of our cohort) included structures beyond just the high EI nodes (like hippocampus and amygdala). A focal therapy such as LITT could provide a more accurate correlation of nodal response to outcome in the future (33). 2) The presence of anti-seizure drugs could have influenced the cortical excitability and response to DCS. However, restarting medications before DCS is commonly practiced to prevent evoked tonic-clonic seizures.

## Conclusion

In the present study, we used DCS to investigate the ictogenicity of putative epileptogenic nodes with high EI values (>0.3). We observed that ictogenicity (i.e., the propensity to induce habitual seizures) of a high EI node is only 44.5%, while 55.4% failed to induce a seizure but had runs of ADs. The LAFA onset pattern had a significantly higher responder rate to DCS (i.e., induced a seizure), while the HYP pattern yielded maximum false responses (i.e., runs of AD's without seizures). The information may be used to support targeted mapping of SOZ in LAFA onset patterns by performing DCS in high EI nodes to distinguish seizure generators (true responders) from hyperexcitable nodes that may be involved in early propagation.

## Data Availability Statement

The data analyzed in this study is subject to the following licenses/restrictions: requests to access the datasets must first be approved by the University of Alabama at Birmingham (UAB) Institutional Review Board (IRB). Requests to access these datasets should be directed to Sandipan Pati, patilabuab@gmail.com.

## Ethics Statement

The studies involving human participants were reviewed and approved by UAB IRB. Written informed consent for participation was not required for this study in accordance with the national legislation and the institutional requirements.

## Author Contributions

AI curated the data, performed analysis, and helped in drafting the manuscript. GC, ET, and DP helped in data analysis and revised the manuscript. SP obtained approval and wrote the manuscript. All authors contributed to the article and approved the submitted version.

## Conflict of Interest

The authors declare that the research was conducted in the absence of any commercial or financial relationships that could be construed as a potential conflict of interest.

## Publisher's Note

All claims expressed in this article are solely those of the authors and do not necessarily represent those of their affiliated organizations, or those of the publisher, the editors and the reviewers. Any product that may be evaluated in this article, or claim that may be made by its manufacturer, is not guaranteed or endorsed by the publisher.

## Abbreviations

EI: epileptogenicity index
DCS: direct cortical stimulation
SOZ: seizure onset zone
EZ: epileptogenic zone
EN: epileptogenic network
TLE: Temporal lobe epilepsy
SEEG: stereo EEG
HYP: hypersynchronous patterns
LAFA: low amplitude fast activity
LITT: laser interstitial thermal therapy.
